# Corticosteroids Contribute to Serious Adverse Events Following Live Attenuated Varicella Vaccination and Live Attenuated Zoster Vaccination

**DOI:** 10.3390/vaccines9010023

**Published:** 2021-01-06

**Authors:** Nathan B. Price, Charles Grose

**Affiliations:** 1 Department of Pediatrics, Division of Infectious Diseases, University of Arizona, Tucson, AZ 85724, USA; pricen@peds.arizona.edu; 2 Department of Pediatrics, Division of Infectious Diseases/Virology Laboratory, University of Iowa, Iowa City, IA 52242, USA

**Keywords:** varicella-zoster virus, severe combined immunodeficiency, natural killer cells, RNA polymerase III, chronic lymphocytic leukemia, HIV, rheumatoid arthritis, prednisone, varicella meningitis, herpes zoster

## Abstract

Corticosteroids, when given in high dosages, have long been recognized as a risk factor for severe infection with wild-type varicella-zoster virus in both children and adults. The goal of this review is to assess the degree to which both low-dosage and high-dosage corticosteroids contribute to serious adverse events (SAEs) following live varicella vaccination and live zoster vaccination. To this end, we examined multiple published reports of SAEs following varicella vaccination (Varivax^TM^) and zoster vaccination (Zostavax^TM^). We observed that five of eight viral SAEs following varicella vaccination, including two deaths, occurred in children receiving corticosteroids, while one of three fatal viral SAEs following live zoster vaccination occurred in an adult being treated with low-dosage prednisone. The latter death after live zoster vaccination occurred in a 70 year-old man with rheumatoid arthritis, being treated with prednisone 10 mg daily. Thus, corticosteroids contributed to more severe infectious complications in subjects immunized with each of the two live virus vaccines. Further, when we surveyed the rheumatology literature as well as individual case reports, we documented examples where daily dosages of 7.5–20 mg prednisone were associated with increased rates of severe wild-type varicella-zoster virus infections in children and adults.

## 1. Introduction

**Figure 1:**
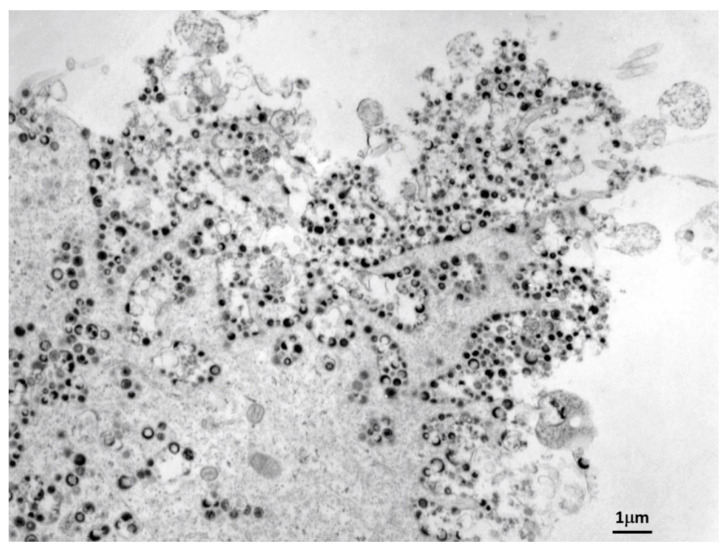
Electron micrograph of cells infected with the varicella vaccine virus. Hundreds of viral particles are easily detected in fibroblast cells infected with the vaccine strain Oka. These infected cells are harvested under non-denaturing conditions in order to produce the live varicella vaccine product. Each vial of varicella vaccine contains 1350 plaque-forming units of infectious viral particles similar in appearance to those seen in this figure.

In this review, we propose that corticosteroids, even when prescribed at what are considered low dosages, increase the risk of a serious adverse event (SAE) in an individual immunized with either live varicella vaccine or live zoster vaccine. The live attenuated varicella vaccine was approved by the Food and Drug Administration in the United States in 1995 [1]. This approval marked the culmination of extensive preclinical and clinical vaccine development over the prior two decades. The attenuation of varicella-zoster virus (VZV) by the traditional method of repetitive passage of virus in cultured cells was first performed in the Takahashi laboratory in the early 1970s; at that time, too little was known about the antigenic viral proteins to develop a subunit vaccine. The review by Takahashi provides a summary of various basic and clinical research studies conducted in Japan and the United States [2]. In that review, Takahashi notes that he was motivated to develop a live attenuated varicella vaccine because of severe cases of varicella that he had seen in hospitalized children. Since that time, all states in the United States have adopted immunization policies that recommend universal varicella immunization of all children [3]. The program has been successful and wild-type varicella (chickenpox) has almost been eliminated as a childhood illness in the United States [4]. The number of deaths from varicella in children has also been sharply reduced [5]. The live attenuated herpes zoster vaccine was approved by the FDA in 2006 [6]. The herpes zoster vaccine is the same attenuated virus as the varicella vaccine, except the number of infectious units was increased by 14 fold [7]. Both live vaccines are prepared from vaccine strain Oka attenuated in the Takahashi laboratory after growth in cultured cells (Figure 1) [2].

The two live attenuated vaccines are now approved by many countries around the world [8,9]. The safety profiles of both vaccines are excellent [10,11]. However, since both are live attenuated vaccines, there will always be a few individuals who have a serious infectious complication because of continued virus replication in the immunized child or adult [2,3]. By SAE, we mean disseminated vaccine varicella infection or death. Several case reports of these SAEs have been published [12]. We recently reported a case of an immunocompetent adolescent who developed varicella vaccine meningitis after a bout of herpes zoster caused by the vaccine virus [13]. After an extensive investigation of her medical history, we discovered that she had been prescribed a short course of low-dose prednisone 4 weeks before her bout of herpes zoster. Because of this case, we decided to review a selected number of the most detailed published reports of SAEs following either live varicella vaccination or live zoster vaccination, to determine how many of these patients had also received corticosteroids. Some of these vaccine recipients did have a previously undiagnosed immune deficiency; nevertheless, the addition of corticosteroids seemed to have worsened the SAE, sometime with a fatal conclusion that may not have occurred in the absence of corticosteroids. We have not found a similar comparative analysis in any prior review of these two vaccines. For example, we note that the authors of a comprehensive 22-year review of safety data about live varicella vaccine from Merck & Company did not distinguish SAEs in children taking corticosteroids from those not taking corticosteroids [14].

## 2. Case Summaries of Viral SAEs after Live Varicella Vaccination

In this section, we provide our summaries of reported cases of a SAE in association with corticosteroids following live attenuated varicella vaccination. Since 1995, over 200 million varicella vaccinations have been administered around the world. Our comments are not a criticism of any therapeutic approach selected by the authors of the cited case reports. We note that there are at least 86 reported fatal SAE cases following live varicella vaccination [12,14]. The published summaries of most of these cases were extremely brief. The main inclusion criterion for this analysis required that the authors state the dosage of corticosteroids if corticosteroids were mentioned in the case summary: in other words, the number of doses per day (in mg or mg/kg) and the number of days that the doses were prescribed. A second inclusion criterion was an adequate description of the entire hospital course, with a list of other medications given to the patient. All case summaries that lacked this information were excluded, for example, cases that just used the single word “chemotherapy” or “steroids” were not included. With these criteria, we selected eight case reports of fatal or near-fatal SAEs that had the longest published descriptions of all the medications given to the patients. All cases were from the USA, except for case 5 (Germany) and case 7 (Australia) (see also Table 1).

1. This child was 13 months old when she developed fever, jaundice, diarrhea and a papular rash [15]. Because of a Coombs-positive anemia, she received both intravenous gamma globulin and high-dosage methylprednisolone (80 mg/day). Over the next three weeks, her skin lesions became eschars. Skin biopsy subsequently revealed intranuclear inclusions typical of herpesviruses, and intravenous (IV) acyclovir therapy was initiated. She later died. Three weeks before her illness she had received her varicella vaccination. Vaccine type varicella was recovered from tissues at autopsy. A genetic analysis detected two mutations in exon 2 of the recombination activating gene 2 (RAG2), but she never had the complete picture of severe combined immunodeficiency (SCID).
2. This 13-month-old girl developed disseminated varicella with shock syndrome and death within 4 weeks of varicella vaccination [16]. During her hospitalization, vaccine virus was recovered from respiratory secretions. Genetic testing uncovered adenosine deaminase deficiency leading to SCID. During her hospitalization, she received both antiviral therapy (acyclovir and foscarnet) and antibacterial therapy (cefotaxime and clindamycin), but death ensued. A medical chart review revealed that she had a history of lung infections and oral candidiasis during the second half of her first year of life. There was no mention of corticosteroid prescriptions.
3. This 13-month old was hospitalized because of suspected sepsis. He had a past medical history of failure to thrive with multiple low grade infections [17]. He had been given varicella vaccine 2 weeks before admission. On initial examination, he had signs of pneumonia and hepatitis. He was intubated and treated with broad spectrum antibiotics. Two weeks after admission (4 weeks after vaccination), he developed a rash and the diagnosis of disseminated varicella vaccine infection was made, based on polymerase chain reaction (PCR) results from skin lesions. After 2 weeks of antiviral therapy with IV acyclovir, he improved enough to remove ventilator support. At this time, he was placed briefly on high-dosage dexamethasone (0.3 mg/kg every 6 h for 10 doses). Within 5 days, his vesicular rash returned and he required additional antiviral treatment. He survived his hospitalization and was subsequently diagnosed with SCID and adenosine deaminase deficiency.
4. This 15-month-old girl had had a prior history of pulmonary infections, global developmental delay, failure to thrive, and profound hypotonia [18]. She had a gastrostomy tube placed at 9 months due to poor feeding. She had been hospitalized several times and had been given corticosteroids during these hospitalizations. One week after varicella vaccination at age 14 months, she was hospitalized for respiratory distress. She again received IV methylprednisolone (3.5 mg every 12 h for six doses) followed by prednisolone (3.5 mg every 12 h for four doses) via g-tube for treatment of the pulmonary distress. On hospital day 14, she was discharged. A rash was noticed soon after discharge, and she was readmitted because of pulmonary distress. Vaccine virus was identified in the skin lesions. She was begun on a low-dose prolonged corticosteroid therapy protocol. Despite concomitant treatment with IV acyclovir (130 mg every 6 h) for varicella vaccine infection, she died. No specific immune deficiency was identified; genetic testing excluded SCID.
5. This 4-year-old girl was undergoing treatment for acute lymphoblastic leukemia [19]. Her therapy regimen is not described in detail but would have included either dexamethasone or prednisone. She was admitted because of rapidly progressive multi-organ failure and acute respiratory distress syndrome. Screening PCR testing detected varicella vaccine DNA in the blood. She was treated with IV acyclovir as well as piperacillin, sulbactam, tobramycin and IV immunoglobulin. The child died of varicella pneumonitis on day 10 of hospitalization. The child had received varicella vaccine 32 days before onset of the current illness. Her chemotherapy had been halted one week before vaccination and restarted one week after vaccination.
6. This 6-year-old boy had a long history of atopy, asthma and eosinophilia [20]. He had received his one and only varicella vaccination at age 1 year. Because of his recurrent allergic episodes, including bouts of asthma, he had received several courses of corticosteroids. At age 6, he presented with evidence of a stroke. The stroke was treated with methylprednisolone followed by oral prednisone (2 mg/kg daily for 3 days) and subsequently prednisolone (1 mg/kg daily for 2 weeks). One month later, he developed additional CNS symptoms and vaccine varicella infection was diagnosed after testing the cerebrospinal fluid by virus-specific PCR. Genetic testing revealed a deficiency of dedicator of cytokinesis 8 (DOCK8). His serum IgE level was 472 IU/mL. He was treated with IV acyclovir and oral prednisolone (2 mg/kg daily for 2 weeks).
7. This 6-year-old boy had a history of severe developmental delay and spastic quadriplegia with epilepsy [21]. He had had no serious infectious illnesses during his first 5 years of life. He had received the measles–mumps–rubella vaccination without a problem. However, 3 weeks after his first varicella vaccination in his 6th year, he developed fever and a rash over his entire body. Later he developed pneumonitis. A diagnosis of disseminated varicella vaccine virus infection was made by PCR testing. He was treated with IV acyclovir, flucloxacillin and clindamycin. The child recovered after prolonged antiviral and antibacterial treatment. An immune assessment discovered a reduction in natural killer (NK) cell activity. There is no mention of corticosteroids in the case report.
8. This 11-year-old had a past history of severe congenital cytomegalovirus infections with intellectual disability [22]. She presented with respiratory distress and bilateral alveolar infiltrates on chest film. She had received a varicella vaccination 5 weeks before the current illness. Vaccine virus was identified by PCR in respiratory secretions. Because of the severity of the respiratory distress, she was given methylprednisolone in addition to acyclovir for treatment of her pulmonary disease and disseminated varicella vaccine infection. She improved on this regimen after 17 days and was discharged, with a prescription for one week of acyclovir tablets. However, she died 10 months after the pulmonary infection. The exact cause of death was unclear, but an embolic episode was suspected. An immune assessment uncovered diminished NK cell activity.

## 3. Reassessment of the above Cases

We propose that corticosteroids may have contributed to more adverse outcomes in infants and children already exhibiting an infectious complication after varicella vaccination. It is known that infants with SCID and adenosine deaminase deficiency may not survive wild-type varicella infection [23]. Currently in the United States, most of these infants are identified by routine neonatal screening assays before they receive any live attenuated vaccines. Among the other subjects in the Table 1, the infant with RAG2 deficiency may have had less severe vaccine-related disease in the absence of corticosteroids, in particular, because the immune deficiency first became apparent around 13 months. RAG2 deficiency leads to a broad spectrum of altered B and T cell functions, with variable responses to viral pathogens [24]. There are two children with NK cell deficiencies, a diagnosis known to be associated with a propensity for more severe herpesvirus infections [25]. The administration of high-dosage corticosteroids to one of the two cases may have contributed to a more serious outcome.

Almost certainly, the child with DOCK8 deficiency would have had a milder vaccine varicella infection in the absence of corticosteroids. DOCK8 deficiency is a recessive form of hyper-IgE syndrome [20]. Although this case is cited in some reviews of adverse events of varicella vaccination, this case is different in that the complication occurred 5 years after vaccination. This case more closely resembles the eight cases of varicella vaccine meningitis that occurred years after a single varicella vaccination [26]. However, his case is the first in this meningitis group known to be associated with recent corticosteroids.

**Figure 2:**
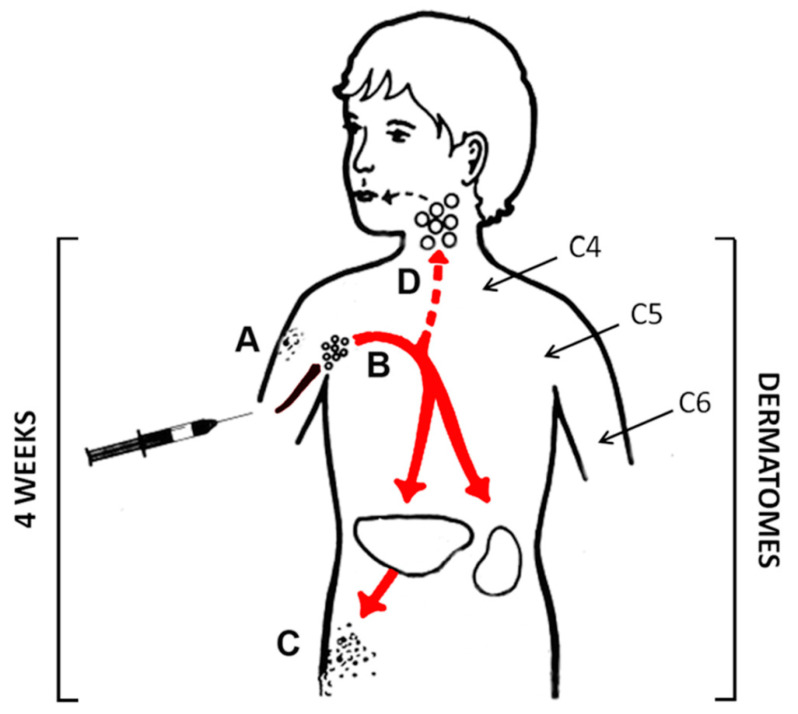
Pathogenesis of varicella virus vaccination. In older children, the vaccine is often administered in the upper arm. After inoculation of the varicella vaccine, the virus replicates locally in the skin for 2–4 weeks (**A**). Some virus is carried in lymphatics to the local lymph nodes. Other viral particles enter the sensory nerves and are carried to the cervical dorsal root ganglia of the spinal cord, where they enter latency (dermatomes C4, C5 and C6). In around 50% of vaccinated immunocompetent children, there is sufficient viral replication in the local lymph nodes, so as to allow for a subsequent viremia (**B**–**D**). The same sequence of events occurs in children who receive their first immunization in the thigh at age 1 year, except that virus enters the inguinal lymph nodes and the lumbar dorsal root ganglia.

Moreover, we can provide an answer to the emergence of disseminated varicella vaccine infection in the child with leukemia [27]. Corticosteroids markedly increase the severity of varicella infections in children with leukemia [28]. The replication cycle of the vaccine virus after inoculation into the skin is certainly longer than one week, perhaps 2–4 weeks (Figure 2). Since chemotherapy was halted for only one week after vaccination, vaccine virus would have continued its replication while the child’s immune response was being suppressed by the chemotherapy regimen [27]. It is important to remember that as many as 50% of healthy children who are first immunized have a viremic phase lasting into the 4th week, in which the virus travels throughout the body [29]. However, in healthy children, an active adaptive immune response prevents any systemic viral SAE.

With regard to children with untreated HIV infection, there are several reports of disseminated varicella vaccine infection after immunization. However, most reports are so brief that we cannot determine if any patient also received corticosteroids [30]. We found one longer report of a 16-month-old boy who was admitted because of fever and respiratory distress [31]. He also had had a one-month history of a progressive erythematous papular rash. He had had poor weight gain since age 6 months. He had received his varicella vaccination at age 13 months. Upon admission, his rash was diagnosed as a vaccine varicella exanthem. Thus, he had a persistent viremia (Figure 2). An HIV test was positive. After initiation of antiviral therapy, his rash disappeared and his general condition improved. He had not received corticosteroids.

## 4. Case summaries of Viral SAEs after Live Zoster Vaccination

In this section, we present three SAEs after live zoster vaccination. The live zoster vaccine is approved in many countries around the world, including the United Kingdom, France, Germany, Italy, India, Australia, Singapore and South Korea [9]. The three cases with SAEs after the live zoster vaccine occurred in three different countries (see Table 2).

1. This case is a 79-year-old man from Scotland with chronic lymphocytic leukemia (CLL) [32]. The patient had received chemotherapy with fludarabine, cyclophosphamide and rituximab in the past, but he had not received any chemotherapy for 6 months. Then, he was given a live attenuated zoster vaccination. Within 2 weeks, he manifested signs of a disseminated zoster vaccine infection. Vaccine virus was identified in the skin vesicles. Even after receiving antiviral treatment, the patient died on day 25 of his hospitalization. There is no mention in the case report that the patient received corticosteroids for any reason during the 6-month interval between cessation of chemotherapy and administration of the zoster vaccine.
2. This case is a 71-year-old man from Australia with CLL [33]. He was not receiving active treatment for CLL because of other chronic illnesses. He was given a live attenuated zoster vaccination 3 weeks before he presented with a bilateral facial rash diagnosed as herpes zoster. Subsequently, he developed signs of a disseminated zoster vaccine infection with meningoencephalitis, confirmed by PCR testing. He died of respiratory failure secondary to varicella pneumonitis. There is no description of treatment with corticosteroids in the case report.
3. This case is a 70-year-old man from Canada with rheumatoid arthritis [34]. He was being treated with methotrexate, hydroxychloroquine and prednisone. The prednisone dosage was 10 mg/day. He had a history of chickenpox as a child. One month after receiving a live attenuated zoster vaccine, he developed a disseminated zoster vaccine infection and died on the fifth day of his viral illness. Vaccine virus was confirmed by PCR testing of a skin vesicle. It is clearly stated in the case report that the patient continued to take prednisone on the day that he was immunized and probably until the day he developed a rash.

## 5. Reassessment of the above Cases

We propose that corticosteroids may be contributing to a more adverse outcome in patients immunized with the live zoster vaccine. Corticosteroids were not mentioned as a risk factor in an earlier safety profile of the live zoster vaccine [11]. The pathogenesis of live zoster vaccination is similar to that following live varicella vaccination, with one exception (Figure 2). More than 90% of older adults who receive live zoster vaccination would have had wild-type varicella as a child. Therefore, >90% of immunocompetent adults would have only local replication of virus in the arm with no viremia. Although vaccine virus replicates locally in the arm for a few weeks, the appearance of an anamnestic adaptive immune response within days of post-immunization limits a viremic spread beyond the arm [35].

Older adults with CLL are known to have a higher risk of wild-type herpes zoster from reactivation of their latent varicella infection as a child [36]. This reassessment confirms that CLL itself and not the chemotherapy is a risk factor for an adverse event after vaccination with the live zoster vaccine (Table 2). One of the two immunized CLL patients had never had chemotherapy and the other had stopped chemotherapy for 6 months, yet both died after being immunized with the live zoster vaccine. Again, based on the pathogenesis model (Figure 2), the virus in immunocompromised CLL patients continues to replicate in the arm and eventually spreads within lymphocytes through the body. The third individual who died due to disseminated vaccine virus infection after zoster immunization had rheumatoid arthritis and was being treated with three medications, including daily prednisone at a low dosage (10 mg/day). For reasons explained in the next section, we consider a dosage of 10 mg/day sufficient to facilitate the fatal varicella infection by impairing the adaptive immune response.

Recently, the Mogensen group in Denmark has discovered that some children and adults with severe varicella-zoster virus meningoencephalitis have a missense mutation in the RNA polymerase III gene [37]. In turn, this mutation leads to deficient interferon production. Although not yet reported, it seems likely that some RNA polymerase-III-deficient recipients of live varicella vaccine or live zoster vaccine would have a severe adverse event.

## 6. Corticosteroids as a Risk Factor for Severe Varicella and Herpes Zoster

After a literature survey in 1993 (before approval of varicella vaccination), the risk of severe wild-type varicella was estimated to be 178 times greater in children receiving corticosteroids when compared to those not receiving corticosteroids [38]. In that survey, most children were receiving what the authors considered to be high-dosage corticosteroids (>2 mg/kg/day of prednisone). As stated in the Introduction, our renewed interest in this subject was motivated by a case of varicella vaccine meningitis that we reported in those who had received lower dosage corticosteroids [13]. The adolescent had received a prescription for a short-term course of prednisone to treat asthma, 4 weeks before her hospitalization for meningitis. She weighed 72 kg and her height was 1.7 m. Even though the prednisone dosage was low (20 mg/day; 0.3 mg/kg/day), the interval between the two events was suggestive. A recently published case of fatal varicella was similar [39]. This 19-year-old girl had just been placed on a dosage of methylprednisolone of 24 mg/day (equivalent to 30 mg prednisone/day) because of a recent diagnosis of lupus. During the first two weeks of this regimen, she contracted wild-type varicella infection and quickly died after dissemination. Although her weight was not given, the dosage was certainly <1 mg/kg/day prednisone.

The association of corticosteroids with disseminated varicella and herpes zoster has been recognized in pediatrics for almost 70 years [40]. Prednisone causes lymphopenia and monocytopenia after ingestion, and thereby abrogates an immune response [41,42]. Dosages of prednisone as little as 10 mg daily can lead to reductions in circulating T cell subpopulations [43]. Both CD4 and CD8 subpopulations are reduced, but there is a disproportionate decrease in the CD4 subpopulation. An informative analysis of immune gene expression in blood cells, measured by an Affymetrix Human Gene 1.1microarray chip, was carried out on 28 adult volunteers who were given 30 mg prednisone daily for 4 days [44]. The investigators documented that short-term prednisone therapy produced a distinct immune signature that included the down-regulation of genes involved in NK cell mediated cytotoxicity. The latter results offer insight into the numerous papers written over past decades that document the increased severity of varicella infection as well as increased likelihood of herpes zoster in both immunocompetent and immunosuppressed children and adults following treatment with corticosteroids [45,46,47,48,49]. One epidemiology study in Denmark that evaluated all cases of herpes zoster that occurred nationwide between 1997 and 2013 found that oral corticosteroids were an independent risk factor [50]. In a similar epidemiology study involving the medical database for Taiwan for the years 2004–2005, investigators determined the oral corticosteroids were a risk factor for herpes zoster in patients with chronic obstructive pulmonary disease, an underlying condition not associated with generalized immune impairment [51].

Because of our particular interest in low-dosage corticosteroids, we have included Table 3 with an emphasis on specific articles [40,49,52,53]. For example, an increased herpes zoster in RA patients is associated with a total dosage as low as 7.5 mg/day [52]. This dosage is lower than that received by the cited rheumatoid arthritis patient with disseminated herpes zoster, included in Table 2. Similarly, increased herpes zoster in patients with lupus is associated with doses equal to or less than 20 mg/day [54]. The latter article made an important observation that 65% of the herpes zoster episodes occurred during mild or inactive lupus when the patients were receiving <20 mg prednisone daily with no other immunosuppressive therapy.

Prescriptions of short-term corticosteroids are now a relatively common practice in the United States, for a variety of acute respiratory, rheumatologic and allergic conditions. According to the Agency for Healthcare Research and Quality, U.S. Department of Health and Human Services, over 25 million prescriptions for prednisone are recorded annually in the United States for both children and adults. There is no consensus as to what should be considered low-dosage corticosteroids, although prednisone dosages of <1 mg/kg/day usually fall into this category.

## 7. Conclusions

The goal of this review is to present data about the effects of corticosteroids on recipients of both the live varicella vaccine and the live zoster vaccine. Although many reports have discussed the effects of corticosteroids on patients with wild-type varicella or herpes zoster, very few reports include children or adults who have received the live attenuated varicella virus. Some of the patients described in this review may have had other comorbidities that contributed to the SAE, for example, obesity, chronic heart or lung disease, smoking and low vitamin D levels. Nevertheless, the recent report that dosages as low as 7.5 mg/day in patients with rheumatoid arthritis led to increased wild-type herpes zoster is very informative (Table 3). The study included over 28,000 patients [52]. The disease rheumatoid arthritis itself is not known to be immunosuppressive; indeed, the authors found that the increased rate of herpes zoster was not associated with degree of rheumatoid arthritis clinical signs or time since onset of rheumatoid arthritis. Furthermore, the previously cited fatal case of disseminated virus infection after live zoster vaccination of an adult with rheumatoid arthritis confirms conclusions about low-dose prednisone in the prior report (Table 4).

One counter-intuitive conclusion of this review is that the Oka strain found in the two live vaccines (Varivax^TM^ and Zostavax^TM^) may be more attenuated than seemingly implied by >80 fatal SAE case reports. This important conclusion is based on the apparent co-administration of corticosteroids along with the vaccines in several case reports with a SAE. Again, based on this review, there is an impression that the immune mechanisms that control varicella replication are more susceptible to suppression by corticosteroids than is recognized in the literature. As an example of lack of awareness, we point out an advisory about administration of the live zoster vaccine by the Public Health Agency in South Korea: ingestion of low-dosage corticosteroids is not a contraindication for administration of the live zoster vaccine [55]. On the other hand, we also note that this review is a retrospective analysis and subject to bias during selection of the case reports.

There is one final question to consider. If corticosteroid usage is so common, could corticosteroids be a cause of herpes zoster in vaccinated children? Currently there is no overall explanation for varicella vaccine reactivation years after immunization [56,57,58]. We propose a two-hit hypothesis. Two major publications have proposed that varicella virus maintains latency by having occasional subclinical reactivations, after which the immune response is boosted; one publication discussed wild-type varicella and the other vaccine-type varicella [59,60]. A third study analyzed peripheral blood mononuclear cells from 99 healthy VZV-seropositive adults by VZV-specific PCR testing and detected wild-type VZV DNA in blood samples from three people [61]. In other words, small amounts of virus are periodically released from the neurons in the dorsal root ganglia and briefly escape immune surveillance. Shortly thereafter they are destroyed by the immune response, which, in turn, is boosted for another extended period. We propose that the clinically apparent reactivation of varicella vaccine virus can occur more easily when, by chance, virus reactivation in the dorsal root ganglia occurs simultaneously with a prescription to the patient for corticosteroids. Based on this literature review, a total daily prednisone dosage of 20 mg or less in either an immunocompetent child or adult is sufficient to allow varicella reactivation. This hypothesis is supported by a guinea pig model for VZV latency, where the animals are injected once with corticotrophin-releasing hormone to induce reactivation [62]. For all the above reasons, we urge more detailed documentation of any corticosteroids prescribed in the 6 weeks preceding a SAE involving either live varicella or live zoster vaccine.

## Figures and Tables

**Table 1:** Serious adverse events in vaccinated children.

Case	Age	Gender	Diagnosis	Steroids	Outcome	Reference
1	13 months	Girl	RAG2	Yes	Survived	[15]
2	13 months	Girl	SCID/ADA	No	Death	[16]
3	13 months	Boy	SCID/ADA	Yes	Survived	[17]
4	15 months	Girl	Tcell	Yes	Death	[18]
5	4 years	Girl	ALL	Probable	Death	[19]
6	6 years	Boy	DOCK8	Yes	Encephalitis	[20]
7	6 years	Boy	NKcells	Unknown	Survived	[21]
8	11 years	Girl	NKcells	Yes	Death	[22]

Abbreviations: m, month; y, year; RAG2, recombination activating gene 2; SCID, severe combined immunodeficiency; ADA, adenosine deaminase; ALL, acute lymphoblastic leukemia; DOCK8, dedicator of cytokinesis 8; NK, natural killer.

**Table 2:** Serious adverse events in immunized adults.

Case	Age	Gender	Diagnosis	Steroids	Outcome	Reference
1	79	Male	CLL	No	Death	[32]
2	71	Male	CLL	No	Death	[33]
3	70	Male	RA	Yes	Death	[34]

Abbreviations: CLL, chronic lymphocytic leukemia; RA, rheumatoid arthritis.

**Table 3:** Lower dosage prednisone followed by disseminated varicella or zoster.

Geography	Age	Condition	Prednisone/Day	Complication	Reference
England	11 years	Competent	20 mg	Disseminated Varicella (WT)	[40]
Brazil	Children	Lupus	20 mg	Herpes Zoster (WT)	[46]
Philippines	Adults	Lupus	20 mg	Herpes Zoster (WT)	[50]
USA	Adults	Lupus	20 mg	Herpes Zoster (WT)	[51]
China	19 years	Lupus	30 mg	Fatal Dissemination (WT)	[39]
USA	Adults	Arthritis	7.5 mg	Herpes Zoster (WT)	[49]
Canada	70 years	Arthritis	10 mg	Fatal Dissemination (VAC)	[34]
USA	14 years	Asthma	20 mg	Meningitis (VAC)	[13]

Abbreviations: WT, wild-type virus; VAC, vaccine-type virus.

**Table 4:** Fatal SAE after live zoster vaccination.

Time Zero: Zostavax	2 Weeks Later	4 Weeks Later	5 Weeks Later
70-year-old man Rheumatoid arthritis Congestive heart failure Methotrexate 25 mg/d Hydroxychloroquine 200 mg/d Prednisone 10 mg/d	Rash on head Rash over body Shortness of breath Chills Continued on the 3 medications	First seen in Emergency department Diagnosis of disseminated varicella Acyclovir treatment begun	Death from disseminated varicella Virus found to be vaccine type Reference [34]
